# Anorexia nervosa and Wernicke-Korsakoff syndrome: a case report

**DOI:** 10.1186/1752-1947-4-217

**Published:** 2010-07-20

**Authors:** Laura Saad, Luiz FAL Silva, Claudio EM Banzato, Clarissa R Dantas, Celso Garcia

**Affiliations:** 1 Department of Psychiatry, Faculty of Medical Sciences, University of Campinas (Unicamp), PO Box 6111, Campinas, SP - Brazil, ZIP code: 13081-970

## Abstract

**Introduction:**

Wernicke's encephalopathy is an acute, potentially fatal, neuropsychiatric syndrome resulting from thiamine deficiency. The disorder is still greatly under-diagnosed, and failure to promptly identify and adequately treat the condition can lead to death or to the chronic form of the encephalopathy - Korsakoff's syndrome. Wernicke's encephalopathy has traditionally been associated with alcoholism but, in recent years, there has been an increase in the number of clinical settings in which the disorder is observed.

**Case presentation:**

We report the case of a 45-year-old Caucasian woman who arrived at the emergency room presenting signs of marked malnutrition and mental confusion, ataxic gait and ophthalmoplegia. Main laboratory test findings included low serum magnesium and megaloblastic anemia. Brain magnetic resonance imaging revealed increased T2 signal in the supratentorial paraventricular region, the medial regions of the thalamus and the central and periaqueductal midbrain. The diagnosis of Wernicke's encephalopathy was made at once and immediate reposition of thiamine and magnesium was started. The patient had a long history of recurrent thoughts of being overweight, severe self-imposed diet restrictions and self-induced vomiting. She had also been drinking gin on a daily basis for the last eight years. One day after admittance the acute global confusional state resolved, but she presented severe memory deficits and confabulation. After six months of outpatient follow-up, memory deficits remained unaltered.

**Conclusion:**

In this case, self-imposed long-lasting nutritional deprivation is thought to be the main cause of thiamine deficiency and subsequent encephalopathy, but adjunct factors, such as magnesium depletion and chronic alcohol misuse, might have played an important role, especially in the development of Korsakoff's syndrome. The co-morbidity between eating disorders and substance abuse disorders has emerged as a significant health issue for women, and the subgroup of patients with anorexia nervosa who also misuse alcohol is probably at a particular risk of developing Wernicke-Korsakoff syndrome. The present case report highlights this relevant issue.

## Introduction

Wernicke's encephalopathy (WE) is an acute, neuropsychiatric syndrome resulting from thiamine deficiency. According to autopsy-based studies, the disorder is still greatly underdiagnosed in both adults and children: the classic triad of WE - the acute onset of ocular abnormalities, ataxia and a global confusional state - is present in only 16% of affected patients [1].

Thiamine (in its converted form: thiamine pyrophosphate) is necessary to several biochemical pathways in the brain, such as intermediate carbohydrate and lipid metabolism and production of amino acids and glucose-derived neurotransmitters (for example, glutamic acid and gamma-aminobutyric acid). Although the disorder has been traditionally associated with alcoholism, any condition of unbalanced nutrition that lasts for 2-3 weeks can lead to thiamine depletion and brain lesions - usually in vulnerable regions, with high thiamine content and turnover, in diencephalic and brainstem areas [1]. In recent years, there has been an increase in the number of clinical settings in which WE is observed. Some of the nonalcoholic conditions associated with this disorder include: prolonged intravenous feeding; hyperemesis gravidarum; anorexia nervosa; refeeding after starvation; thyrotoxicosis; malabsorption syndromes; hemodialysis; peritoneal dialysis; AIDS; malignancy; and gastroplasty with postoperative vomiting [1].

WE remains largely a clinical diagnosis. No specific diagnostic abnormalities have been found in cerebrospinal fluid, brain imaging or electroencephalograms [1]. Although the presumptive diagnosis of WE can be confirmed by assessing the thiamine status by the direct measurement of thiamine pyrophosphate in erythrocytes, whole blood by high performance liquid chromatography [2] or by performing the erythrocyte transketolase activation test or the transketolase activity assay [3], these measurements are limited by low specificity and technical difficulty [1]. Even though a new chromatography method for the assessment of thiamine, thiamine monophosphate and thiamine diphosphate in human erythrocytes - which seems to be more suitable for clinical and research purposes than previous methods - has been more recently described [4], it is still unavailable in most clinical settings. Magnetic resonance Imaging (MRI) is currently considered the most valuable method for confirming a diagnosis of WE. MRI has a sensitivity of only 53%, but high specificity: 93%. MRI studies typically show an increased signal in T2 and fluid-attenuated inversion recovery (FLAIR) sequences, bilaterally symmetrical in the paraventricular regions of the thalamus, the hypothalamus, mamillary bodies, the periaquedutal region, the floor of the fourth ventricle and midline cerebellum. However, the typical pattern of lesions on MRI is observed in only 58% of patients. Unusual sites of lesions include cortical regions and the splenium of the corpus callosum [5].

WE is a medical emergency. In patients for whom the disorder is suspected, thiamine should be initiated immediately in order to prevent irreversible brain damage which can lead to death, with an estimated mortality rate of about 20%, or to the chronic form of the encephalopathy - Korsakoff's syndrome - in up to 85% of survivors [1]. Korsakoff's syndrome (KS) is characterized by a disproportionate impairment in memory, relative to other features of cognitive function. Severe anterograde amnesia is present and usually memory of events in the weeks or months before the disorder is disturbed. Confabulation accompanies the memory loss especially in the early stages. Disorientation of time is noticeable. Other cognitive functions are either preserved or may show only minor deficits (for example, in executive functions). KS usually follows or accompanies WE, with the typical clinical pattern emerging when the acute global confusion state of the latter resolves [1,6].

There have been a few case reports of WE in anorexia nervosa (AN) in literature [7-9]. In most reported cases, clinical signs resolved after thiamine treatment [8,9]. We present a case in which AN, probably mediated by alcohol abuse and magnesium depletion, had led not only to the acute WE but also to the chronic memory impairments characteristic of Korsakoff's syndrome.

## Case presentation

**Figure 1:**
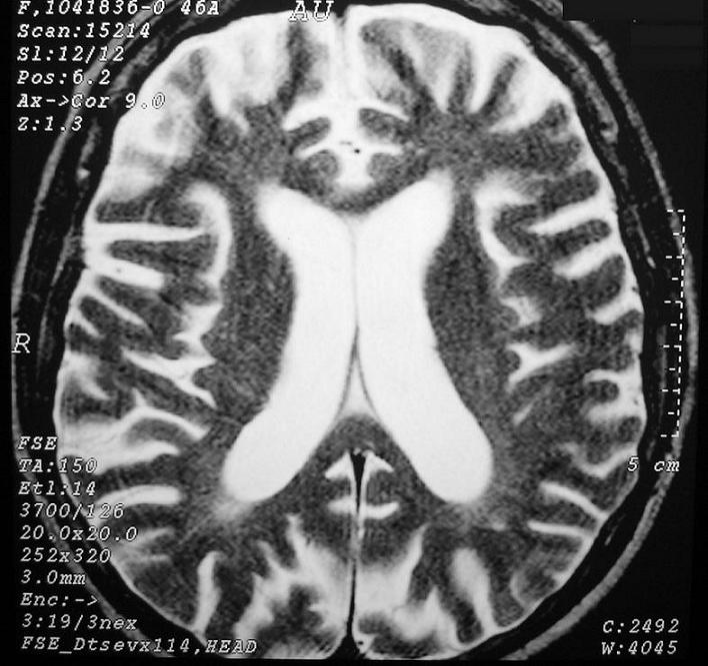
**Brain axial T2W magnetic resonance imaging at the level of the lateral ventricles**. Areas of high signal involving the periventricular white matter associated with enlargement of the lateral ventricles and widening of cerebral sulci.

**Figure 2:**
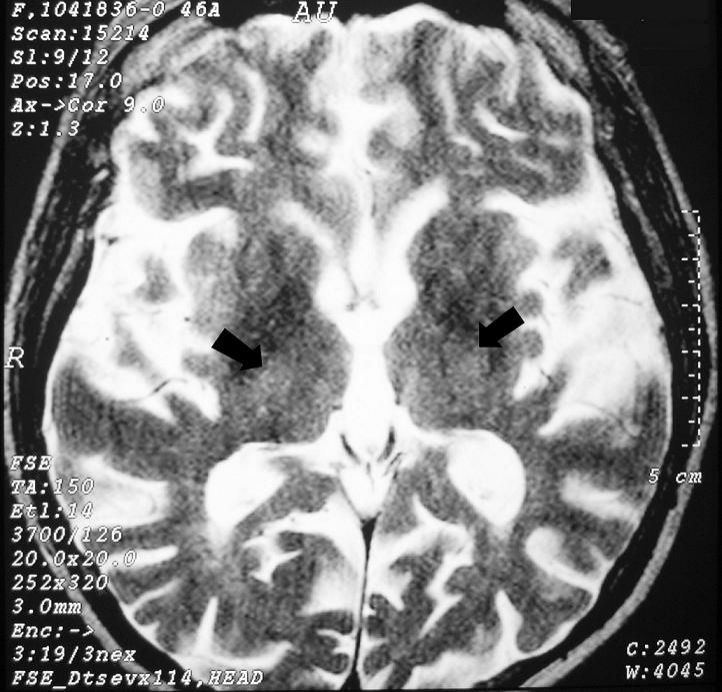
**Brain axial T2W magnetic resonance imaging at the level of the thalami and third ventricle**. High signal foci in the medial regions of thalami (arrows).

**Figure 3:**
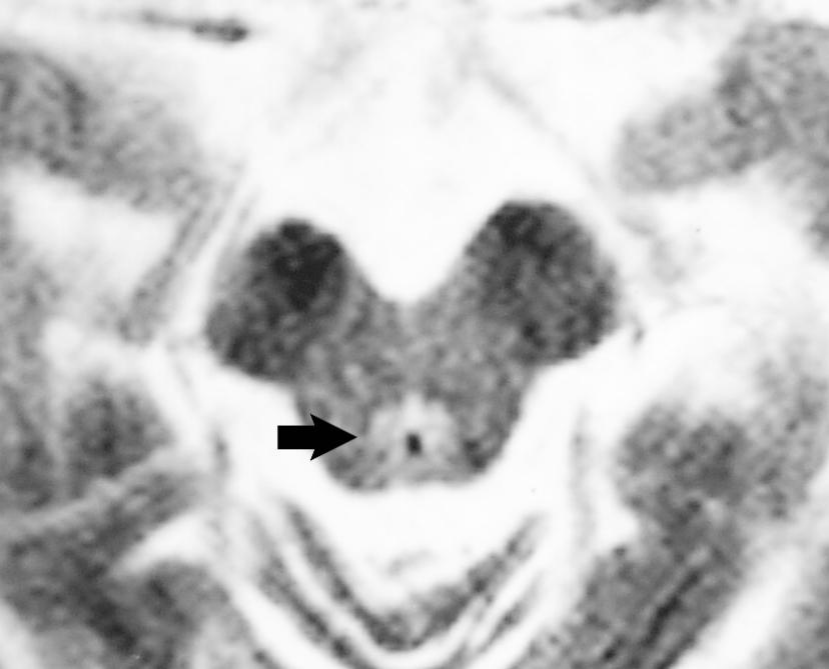
**Brain axial T2W magnetic resonance imaging at the level of the midbrain**. High signal around the Aqueduct of Sylvius (arrow).

**Figure 4:**
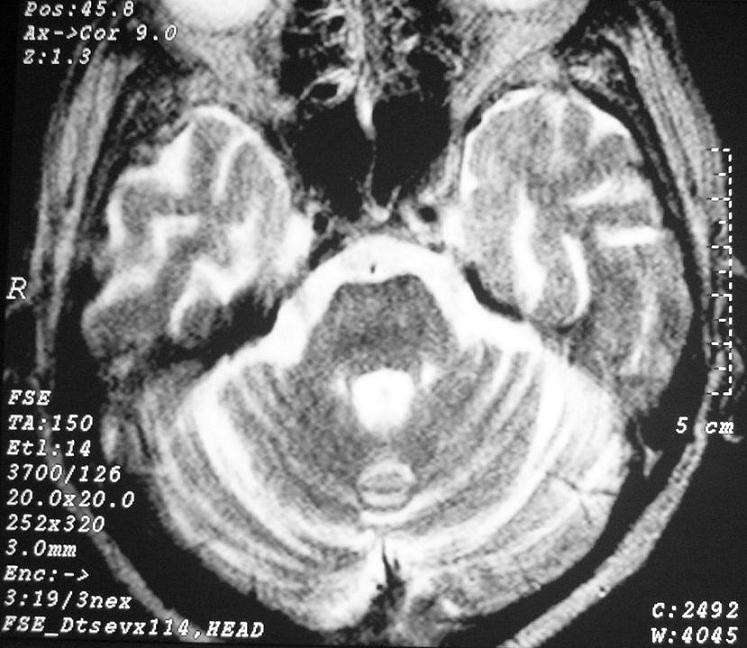
**Brain axial T2W magnetic resonance imaging at the level of the pons**. Widening of cerebellar sulci.

The patient was a Caucasian 45-year-old, non-smoker, single woman with one son. She had been a model when a teenager but was currently unemployed, arrived at the emergency room presenting mental confusion, ataxic gait and ophthalmoplegia (sixth-nerve paralysis), without any other neurological focal sign. Her physical examination showed marked malnutrition and paleness. Her body mass index was 16.42 (height: 1.58 m; weight: 41 kg). Cerebrospinal fluid analyses were normal. A brain MRI (performed the next day) showed, at T2, a thin hypersignal halo in the supratentorial periventricular region, increased T2 signal in the medial regions of the thalamus and in the central and periaqueductal midbrain. There were enlarged perivascular spaces in the basal nuclei topography, a thinned corpus callosum and widened cerebellar sulci (Figures 1, 2, 3, 4). A hemogram revealed megaloblastic anemia: hemoglobin = 10.0 g/dL (normal range (NR) = 12-16 g/dL) with mean corpuscular volume = 113.5 fl (NR = 81.0-99.0 fl), mean corpuscular hemoglobin = 38.5 pg (NR = 27-32.0 pg), red cell size distribution width = 14.5% (NR = 10-15%), red blood cell count = 2.6 × 10^6^/L (NR = 4.2-5.4 × 10^6^/L); folic acid = 2.51 ng/mL (NR = 3.2-17.0 ng/dL), B12 = 300.1 pg/mL (NR = 202-900 mg/dL). The key findings of the serum laboratory tests run were: urea = 102 mg/dL (NR <50 mg/dL); albumine = 2.9 mg/dL (NR = 3.4-4.8 g/dL); triglycerides = 86 mg/dl (NR <150 mg/dL); low-density lipoprotein = 76 mg/dL (NR <100 mg/dL); high-density lipoprotein = 17 mg/dL (NR >40 mg/dL); magnesium = 0.47 mg/dL (NR = 1.3-2.1 mEq/L); and phosphorus = 2.3 mg/dL (NR = 2.7-4.5 mg/dL). The results of the following tests were found to be within the limits of normality: sodium; potassium; creatinine; glucose; amylase; lipase; alanine and aspartate aminotransferase; gamma-glutamyl transpeptidase; alkaline phosphatase; and blood coagulation. The results of serologies for HIV, hepatitis B and C were negative.

The diagnosis of WE was made at once and immediate reposition of magnesium (2 g of magnesium sulfate dissolved in 1 L of normal saline given by infusion over a period of 24 h) and thiamine (300 mg of thiamine hydrochloride, dissolved in 100 mL of normal saline, given by infusion over a period of 30 min, three times a day) was started. After 3 hours the patient presented a sudden decrease in the level of consciousness (Glasgow coma score fell from 15 to 10), with pO2 saturation of 99%, blood pressure of 110 × 70 mmHg, serum glucose 73 mg/dL and no hydroeletrolytic imbalance. The patient did not present with cardiac dysrhythmia, dyspnoea, cutaneous rash or flushing. General support measures were maintained and the patient remained under close observation.

The patient's mother and son informed us that she has been dieting since she was 30 years old. The patient's goal was to weigh 30 kg and this goal was easily reached. From then on, she would never allow herself to weigh over 40 kg. Periods of amenorrhea were common. Recurrent thoughts about actually being fat led her to severely restrict the diet. Every 40 days she spent 10 days vomiting everything she ate and drank. Such information allowed us to diagnose anorexia nervosa of the binge eating/purging type.

In addition, her relatives informed us that the patient had been drinking gin on a daily basis for the last eight years. They told us that she drank about a bottle of gin every three days (approximately 300 mL of gin, or 15 alcohol units, per day). There was no evidence that use of alcohol had escalated over the years and she had never tried to quit or cut back the amount she drank. Besides a certain moodiness, there were no other alcohol withdrawal symptoms. Likewise, in the emergency room she did not present any signs of alcohol withdrawal.

She was admitted to the psychiatric ward. One day after admission her acute global confusional state resolved, but she presented severe memory deficits and confabulation. Psychiatric hospitalization lasted for a month, during which a daily reposition of 600 mg of thiamin was maintained. Magnesium replacement was made with 450 mg/day of magnesium oxide orally and magnesium sulfate (4 g/d for the first three weeks and 1 g/d in the last week). In a neuropsychological battery (the CAMCOG [10]) performed two days before discharge her scores were: orientation - 8 out of 10; language comprehension - 11 out of 11; language expression - 19 out of 19; remote memory- 2 out of 6; recent memory - 4 out of 7; learning memory - 12 out of 20; attention - 18 out of 18; praxis - 2 out of 2; calculation - 2 out of 2; abstract thinking - 5 out of 8; perception - 10 out of 13; and she scored 27 out of 30 in the Mini Mental State Examination. The patient maintained confabulation and still had ataxia. Ophthalmoplegia has resolved. After six months of out-patient follow up, memory deficits remained unaltered.

## Discussion

WE is considered to be a rare complication of AN [11]. In the case presented, clinical suspicion was aroused as a result of the presence of the classical triad of WE. Encephalopathy might be underdiagnosed in AN as WE might have a non-specific clinical presentation and about 19% of patients have none of the symptoms of the classic triad [1]. The prevalence of thiamine deficiency in AN may be as high as 38%, with 19% of patients meeting the most stringent published criterion for deficiency [12]. Meanwhile, some sort of neurological complication is present in up to 45% of AN cases [11].

In this case, self-imposed long-lasting nutritional deprivation is thought to be the main cause of thiamine deficiency and WE, but adjunct factors might have played an important role, especially in the development of KS: magnesium (Mg) depletion and chronic alcohol misuse. Mg as a co-factor has a crucial role in the proper catalytic action of thiamine pyrophosphokinase in the conversion of thiamine into thiamine pyrophosphate. Mg depletion aggravates thiamine deficiency fostering the development of WE [1]. Alcohol ingestion (which causes increased excretion of Mg) and excessive vomiting might have led to Mg depletion.

The patient did not present with some of the crucial features of alcohol dependence syndrome but she maintained a pattern of high ingestion of alcohol for many years. The metabolism of alcohol raises the demand for thiamine, at the same time alcohol decreases the amount of thiamine transported across the intestinal mucosa and impairs the conversion of thiamine to thiamine pyrophosphate [1]. Data indicate that patients who develop WE in association with alcohol misuse are more likely to develop KS than those who do not misuse alcohol, with ethanol neurotoxicity being a possible contributing factor [6].

The patient suffered a sudden decrease in the level of consciousness a few hours after thiamine reposition was started. That raised the question of whether the treatment had an adverse effect on her. Although this hypothesis could not be ruled out, we believe that such an event was part of the consciousness alterations seen in WE, since the patient had no additional sign of an anaphylactic or anaphylactoid reaction. To date, there is no definite consensus on the optimum dose, frequency, route or duration of thiamine treatment for WE but a recent extensive revision on WE published in the journal *Lancet Neurology *[1] recommended even larger doses than we used: 500 mg of thiamine hydrochloride (dissolved in normal saline), given by infusion over 30 min. One limitation of our case report is the lack of follow up neuroimaging, since MRI findings are reversible and can be normalized within a few days after thiamine administration, in conjunction with a symptomatic improvement [5].

In the last decade, the co-morbidity between eating disorders and substance abuse disorders has emerged as a significant health issue for women. Patterns of association vary across eating disorder subtypes. In a review of 25 studies, Holderness *et al. *[13] calculated a median prevalence of 22.9% of alcohol abuse or dependence in women with bulimia nervosa in clinical samples. The majority of studies suggest that the prevalence of alcohol abuse or dependence in women with AN and binge eating (DSM-IV criteria) may be comparable to, or exceed, that observed in women with normal-weight bulimia nervosa. In a multicenter study, Bulik *et al. *[14] reported 37.8% of prevalence of alcohol abuse or dependence in patients with AN/binge eating. The subgroup of patients with AN who also misuse alcohol is probably at a particularly high risk of developing WE and its complication, KS. The present case report highlights this relevant issue.

## Conclusion

There is a significant co-morbidity between eating disorders and substance related disorders and the subgroup of patients with AN who also misuse alcohol is probably at a particularly high risk of developing Wernicke-Korsakoff syndrome. Highlighting this issue, we presented a clinical case in which AN, combined with magnesium depletion and chronic alcohol misuse, had led to the presentation of the complete Wernicke-Korsakoff syndrome.

## Abbreviations

AN: anorexia nervosa; Mg: magnesium; MRI: magnetic resonance imaging; NR: normal range; WE: Wenicke's encephalopathy.

## Consent

Written informed consent was obtained from the patient for publication of this case report and accompanying images. A copy of the written consent is available for review by the Editor-in-Chief of this journal.

## Competing interests

The authors declare that they have no competing interests.

## Authors' contributions

LS and LFALS were responsible for the patient's clinical care and the first draft of the case report section. CRD and CGJ were major contributors in writing the manuscript. CEMB was responsible for the clinical care supervision, coordinated the whole publication effort and reviewed all manuscript versions. All authors read and approved the final manuscript.
